# Tartary buckwheat flavonoids relieve the tendency of mammary fibrosis induced by HFD during pregnancy and lactation

**DOI:** 10.18632/aging.203752

**Published:** 2021-12-10

**Authors:** Xingchi Kan, Juxiong Liu, Xiangyu Cai, Yaping Huang, Ping Xu, Shoupeng Fu, Wenjin Guo, Guiqiu Hu

**Affiliations:** 1 Department of Theoretic Veterinary Medicine, College of Veterinary Medicine, Jilin University, Changchun, Jilin, China

**Keywords:** high-fat diet, inflammatory microenvironment, AKT/NF-κB, blood-milk barrier, fibrosis

## Abstract

Mammary gland fibrosis is a chronic and irreversible disease. Tartary buckwheat flavonoids (TBF) are a natural product of flavonoid extracts from buckwheat and have a wide range of biological activities. The purpose of this experiment was to explore whether HFD during pregnancy and lactation induces fibrosis of the mammary tissue and whether TBF alleviates the damage caused by HFD, along with its underlying mechanism. The HFD significantly increased the levels of TNF-α, IL-6, IL-1β, and MPO; significantly damaged the integrity of the blood-milk barrier; significantly increased the levels of collagen 1, vimentin and α-SMA, and reduced the level of E-cadherin. However, these effects were alleviated by TBF. Mechanistic studies showed that TBF inhibited the activation of AKT/NF-κB signaling and predicted the AKT amino acid residues that formed hydrogen bonds with TBF; in addition, these studies not only revealed that TBF promoted the expression of the tight junction proteins (TJs) claudin-3, occludin and ZO-1 and inhibited the activation of TGF-β/Smad signaling but also predicted the Smad MH2 amino acid residues that formed hydrogen bonds with TBF. Conclusion: HFD consumption during pregnancy and lactation induced the tendency of mammary fibrosis. TBF alleviated the tendency of mammary fibrosis by inhibiting the activation of AKT/NF-κB, repairing the blood-milk barrier and inhibiting the activation of TGF-β/Smad signaling.

## INTRODUCTION

During pregnancy and lactation, due to the high nutrient requirements of the body and the vigorous energy metabolism of the mammary glands, mammary tissues are under tremendous physiological metabolic pressure during pregnancy and lactation [1]. In this state, the mammary epithelial cells are very sensitive and fragile, which tends to cause cell physiological dysfunction or even severe disease [2, 3]. In addition, food intake during lactation is relatively large, and many high-fat foods are often consumed. A high-fat diet (HFD) refers to foods containing many fatty acids, of which saturated fatty acids occupy a high proportion [4]. Studies have shown that long-term intake of HFD was bad for health. Long-term intake of a HFD activates the NF-κB signaling and promotes the accumulation of inflammatory mediators such as IL-1β, IL-6 and TNF-α, which induces the body to produce a long-term chronic inflammatory microenvironment in the body [1, 5], and induces the occurrence of chronic diseases, such as inflammatory bowel disease [6] and mastitis [1], etc. However, whether long-term intake of HFD during pregnancy and lactation will causes an increased likelihood of mammary fibrosis, as well as determining ways how to alleviate chronic mammary diseases, and how to protect mammary health, are questions that urgently need to be addressed urgently.

The blood-milk barrier is an important biological barrier of the mammary, and the integrity of the blood-milk barrier is an important guarantee for its normal physiological functions (Ziv and Heavner, 1984). The tight junction structure is the main component of the blood-milk barrier, which exists in the gap between epithelial cells and controls the flux of paracellular cells [7]. Scaffold proteins (such as ZO-1) and transmembrane proteins (including members of the occludin and claudin families) are the main components of tight junctions [7]. Damage to the blood-milk barrier will reduce the mammary gland's anti-infection ability against pathogenic microorganisms, promote long-term exposure of the mammary gland to unfavorable factors, and increase the risk of mammary gland exposure (Zhao and Lacasse, 2008). In addition, the destruction of tight junction structures is also closely related to the occurrence of fibrotic diseases. Research findings indicate that changes in the epithelial barrier may be important for the pathogenesis of idiopathic pulmonary fibrosis [7]. Studies have shown that the integrity of the lung epithelial tight junction structure was damaged by continuous intraperitoneal injection of bleomycin for 14 days, and the lung tissue showed a tendency to fibrosis [8]. Damage to tight junctions leads to the loss of cell polarity and induces the epithelial mesenchymal transition, which is a major player in fibrosis [9]. In addition, our previous research results indicate that a high-fat diet during pregnancy can destroy the blood-milk barrier (Guo et al., 2020). However, whether a high-fat diet during pregnancy and lactation can induce mammary fibrosis by destroying the blood-milk barrier has not yet been reported.

Fibrosis is a phenomenon of tissue and organ hyperplasia. Sclerotic scar tissue formation and extracellular matrix (ECM) accumulation are the main characteristics of tissue fibrosis. Collagen is the main component of ECM. [10, 11]. Chronic inflammation, autoimmune reaction, tissue damage, chemical stimulation and other factors are the main causes of fibrosis [10, 11]. The expression level of TGF-β1 in fibrotic tissue is significantly higher than that in normal tissue [11]. Smad 3 is an important transcription factor that promotes fibrosis which is regulated by TGF-β1 signals [12]. Studies have shown that inhibiting the activation of TGF-β/Smad is an important target for inhibiting the occurrence of fibrosis [13]. At present, there are relatively few studies on mammary fibrosis, and research on this it is still in its infancy. There are still many important unknown scientific problems waiting to be explored.

Tartary buckwheat flavonoids (TBF) are a biologically active ingredients extracted from the food buckwheat, which mainly contains natural flavonoids [14]. In this study, TBF mainly contained 36.3% rutin and 58.2% quercetin. Studies have shown that rutin significantly improves renal interstitial fibrosis in rats with obstructive nephropathy [15]. Quercetin is the aglycone form of rutin, which also has similar pharmacological functions [14]. Studies have shown that quercetin can relieve hepatitis and fibrosis [16]. Based on the above clues, we speculate that TBF flavonoids are beneficial for mammary health protection. Therefore, the purpose of this experiment was to explore whether a HFD during pregnancy and lactation can induce fibrosis of mammary tissue and to determine whether TBF can alleviate the damage caused by a HFD during pregnancy and lactation to the mammary gland, along with its underlying mechanism. This experiment provides a reference for buckwheat base flavonoids in the diet and for the treatment of HFD-induced mammary diseases.

## MATERIALS AND METHODS

### Animal

Thirty-eight-week-old ICR mice (10 male mice, 20 female mice) were purchased from Changsheng, Liaoning. The mice were divided into cages at a ratio of 2 female mice to 1 male mice in each cage. After the female mice were pregnant, the male mice were removed, and the mice were free to eat and drink. The experimental guidelines were strictly followed during the experiment [17, 18].

### Construction of a mouse model

A HFD (D12492, Xiaoshuyoutai, Beijing, China) containing 60% fat was used to construct the experimental model. The HFD was started after 3 d of pregnancy, and one week after delivery the mammary tissue was collected. The mice were allowed to eat and drink freely and were provided 12 h of light and 12 h of darkness every day.

### Extraction and purification of Tartary buckwheat flavonoids (TBF)

Tartary buckwheat powder was obtained from Nanjing Daosifu Biotechnology Co., Ltd (Nanjing, China). Three volumes of methanol and one volume of water were mixed and refluxed for 2 h. The above operations were repeated 3 times. The methanol extract was concentrated in vacuum and then freeze-dried. Deionized water was used to dilute the extract powder, and then the diluent containing the drug was filtered through a glass column of AB-8 resin for purification.

When the adsorption column reached saturation, it was diluted with 60% ethanol. The collected eluent was successively lyophilized and purified to obtain a buckwheat alkaloid extract. The amounts of rutin and quercetin present in the TBF extract were 10.89 and 17.46 g, respectively, which accounted for up to 94.5% of the TBF (results determined by HPLC). The specific content of TBF is shown in Tables 1–3.

**Table 1:** Main active components of Tartary buckwheat flavonoids (TBF).

**Analysis**	**Specification**	**Results**
Assay (By UV)	Rutin >30%	36.3%
Assay (By UV)	Quercetin >55%	58.2%

**Table 2:** Physical and chemical control of TBF.

**Item**	**Identification**	**Verified**
Appearance	power	Complies
Color	Light Yellow	Complies
Odor	Characteristic	Complies
Taste	Characteristic	Complies
Sulphated Ash	<2%	1.30%
Heavy Metals	<10ppm	Complies
Pesticides	Negative	Negative
Mesh Size	80mesh	Complies
Loss on Drying	<5%	1.9%

**Table 3:** Microbiological index of TBF.

**Item**	**Identification**	**Verified**
Total Plate Count	<1000/g	Complies
Yeast and Mold	<100/g	Complies
Salmonella	Negative	Negative
E. coli	Negative	Negative

TBF (1 g/L) was dissolved in 0.1% carboxymethylcellulose sodium solution (CMC-Na) and was ingested by mice by drinking water. Drinking water administration began on the first day of mating and stopped one week after delivery.

### H&E staining

Fresh mice mammary gland tissue was collected, avoiding the larger blood vessels of the mammary gland, and then subblocks of mammary gland tissue were immersed in 4% formaldehyde solution for 48 h. Then the mammary gland tissue blocks were dehydrated, transparent, impregnated with wax, embedded, sliced and dried to obtain paraffin sections. The results of H&E (G1005, Servicebio, Beijing, China) staining were obtained by xylene dewaxing, gradient alcohol dehydration, hematoxylin and eosin staining and sealing.

The scoring criteria of the mammary gland were the infiltration of inflammatory cells in the mammary acini, the thickness of the mammary matrix, and the dehydration of mammary acini. The specific scores were as follows: 0: no injury; 1: mild injury; 2: moderate injury; 3: severe injury; 4: excessive injury, as previously described [19].

### Masson

Paraffin sections of mammary gland were dewaxed and stained with haematoxylin. After Masson staining, Ponceau staining, molybdophosphoric acid washing, 95% alcohol dehydration, neutral gum sealing and other experimental steps, the final Masson staining results were obtained. The experimental steps strictly follow the instructions (Solarbio, G1340, Beijing, China).

### Western blot

500 μL of protein lysis solution RIPA (Biyuntian Biotechnology, Shanghai, China) was added to 2 mL centrifuge tube containing mice mammary glands of mice. The mice mammary gland tissue was ground for 10 min, and the condition was 50 HZ. Immediately after centrifugation at 12000 revolutions per minute (rpm) for 10 min, the supernatant was obtained and the protein concentration was determined using BCA Protein Assay Kit (Shanghai, China).

12% and 4% SDS-page gels were prepared and used to separate protein samples at 70 V 30 min, 100 V 60 min. Then the PVDF membrane was covered on SDS-page gels containing total protein, and the experimental conditions were 75 V and 90 min. About 100 μL of the first antibody (1:1000) was dripped on the surface of PVDF membrane, and then the PVDF membrane was put into a wet box and kept overnight at 4°C. The first antibody was washed off by PVDF on the next day, and then the second antibody (1:5000) was added to the surface of PVDF membrane. After 1 h, the second antibody on the surface of PVDF membrane was washed off. ECL plus (p1050) was added on the surface of PVDF film for 3 min. Finally, the experimental results were obtained by X-ray exposure in the dark room. Detailed steps are described earlier [20].

Primary antibodies AKT, p-AKT, p-P65, P65, IκB, p-IκB and β-Tubulin were purchased from Cell Signaling Technology, Inc. (Danvers, MA, USA); Collagen 1, E-cadherin, Vimentin were purchased from Affinity Biosciences LTD (Affinity Biosciences, OH, USA). Secondary antibodies (mice source and rabbit source) were purchased from Boster Biological Technology co., ltd. (Hubei, China).

### Determination of the integrity of the blood-milk barrier

2 mg/mL albumin-fluorescein isothiocyanate conjugate (FITC) solution (Sigma-Aldrich, MO, USA) was prepared in advance with PBS. Then about 1 cm^3^ of fresh mammary gland was immersed in FITC solution for 10 min. The mammary gland was quickly put into a thermos cup filled with liquid nitrogen. After the mammary gland was fixed, the mammary gland was taken out, and the mammary gland was put into a −20°C frozen section machine to obtain 5 μm thick frozen sections. About 40 μL of DAPI (Biyuntian Biotechnology, Shanghai, China) was added to the frozen sections, and the results were obtained under the fluorescence microscope.

### Molecular modelling

The 3D structures of AKT and Smad 3 MH2 domains were obtained from the PDB database with numbers 3ow3 and 1MJS, respectively. The 2D structure of the main components of TBF, quercetin and rutin, was obtained from PubChem. The 2D structure was converted into a 3D structure by PyMOL software and stored in pdbqt format. The AutoDock 4 software was used for molecular docking simulation of small molecules and proteins, and 2.5 × 10^6^ calculations are executed. After calculation, the total binding energy of small molecules with proteins and the acting hydrogen bonds are obtained. Finally, use PyMOL software to make a 3D model picture of the hydrogen bonds between small molecules and proteins.

### Determination of MPO and ELISA

4000 μL of HEPES solution was added to 1 g of fresh mammary gland, ground at 50 HZ for 10 min, centrifuged at 12000 rpm for 10 min, and the supernatant was taken as the ELISA test sample. The precipitate of mammary gland was added to the same volume of 0.25% CTAC solution, ground and centrifuged as in the previous article, and the supernatant was taken as the MPO sample for detection. The specific detection methods of MPO and ELISA (Beijing Solarbio Science and Technology Co., Ltd.) are as described in the previous article [21].

### Data and statistical analysis

ImageJ software was used to count the relative density of protein bands, merge the fluorescence result pictures and add scale bar. GraphPad Prism 8 software was used for histogram statistics and data difference analysis. Adobe Illustrator CS6 software was used for group pictures of experimental results. The AutoDock 4 software was used to calculate the molecular simulation between TBF and protein. PyMOL was used to make 3D pictures between TBF and protein. The experimental results are expressed as the mean ± SEM, and all the experimental results are repeated three times independently. The difference between the data was analyzed and analyzed by the one-way ANOVA method.

### Data availability statement

All relevant data about this research can be requested from the corresponding author.

## RESULTS

### Effect of TBF on mammary gland tissue of mice induced by a high-fat diet (HFD) during pregnancy and lactation

We initially explored the effect of a HFD on mammary gland damage during pregnancy and lactation, and whether intake of TBF has a relieving effect on the mammary gland. We observed the mammary gland to evaluate the state of the mammary gland and further performed H&E staining and Masson staining to assess the pathological damage of the tissue.

**Figure 1:**
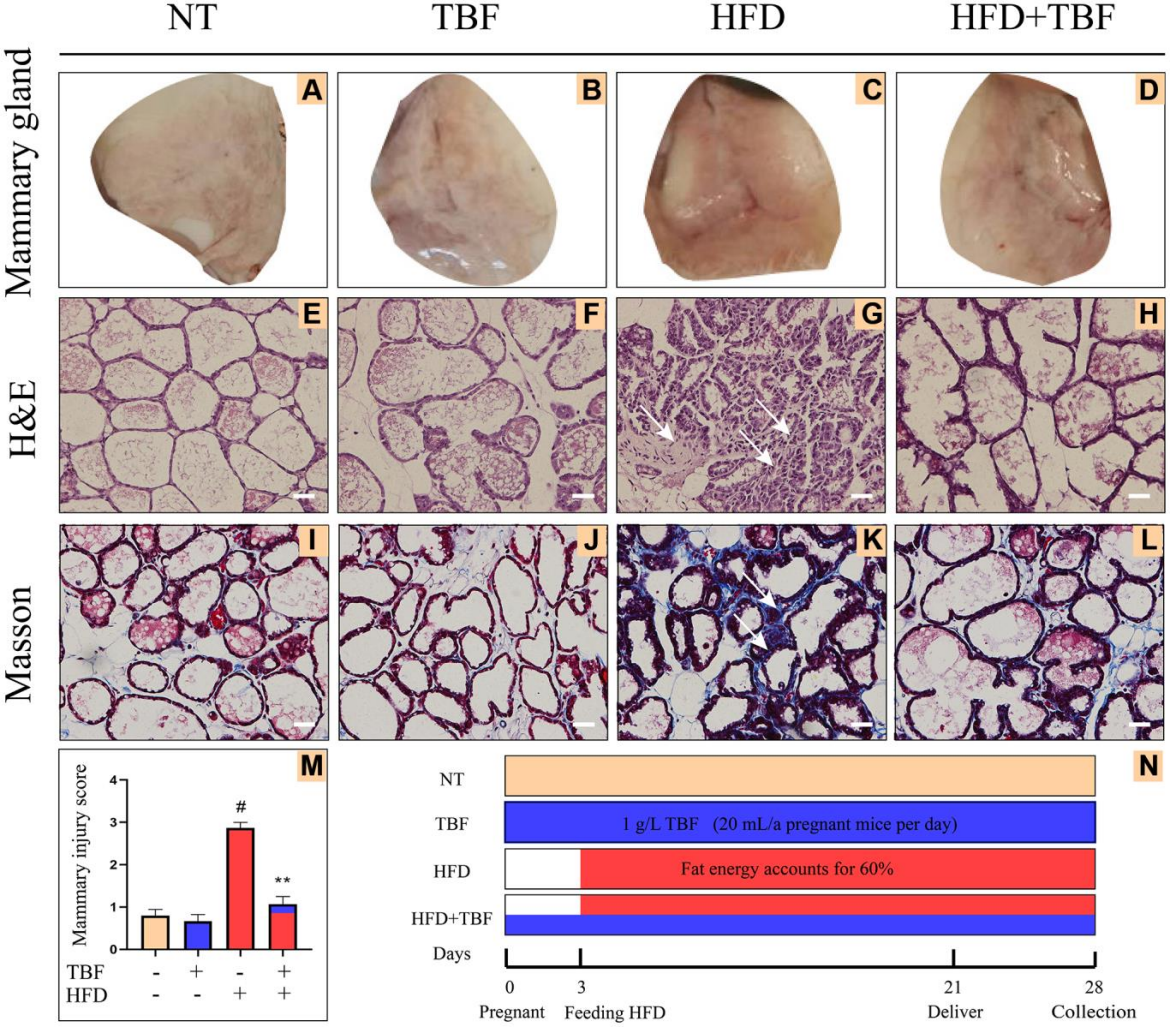
**Effects of Tartary buckwheat flavonoids (TBF) on mammary gland tissues of mice induced by high fat diet (HFD) during pregnancy and lactation.** 1 g/L TBF was dissolved in 0.1% carboxymethylcellulose sodium solution and was ingested by mice by drinking water. Drinking water administration began on the first day of mating and stopped one week after delivery, and then mammary tissue was collected. The mice mammary tissue was made into paraffin sections, which were used for H&E staining and Masson staining. (**A**–**D**) Mice mammary tissue; (**E**–**H**) H&E staining result of mammary tissue; (**I**–**L**) Masson result of mice mammary, blue represents collagen fiber, red represents muscle fiber; (**M**) Mammary gland damage score; The arrow represents the lesion of mammary tissue; (**N**) Schematic diagram of the mice experiment cycle; The arrow represents the lesion of mammary tissue; H&E and Masson are the results of 200× magnification, Scale bar: 100 μm. The data error was based on SEM, three independent repeated experiments were performed; ^#^*p* < 0.01 vs. No treatment group (NT) group; ^∗∗^*p* < 0.01 vs. HFD group.

From the evaluation of the histomorphological results, our experimental results showed that compared with the NT group (no treatment) and TBF group, the mammary tissue of the HFD group was red, and the blood vessels were congested and swollen, indicating that the mammary tissue had a certain degree of inflammation (Figure 1A–1C). However, it was surprising that the drinking beverages containing TBF significantly alleviated these phenotypes described above (Figure 1C, 1D).

From the results of the H&E staining, our experimental results showed that compared with the NT group and TBF group, the mammary gland acini of the HFD group were significantly atrophied, the stroma between the acini was thickened, there was fibrous scar tissue, and the mammary gland tissue was significantly damaged (Figure 1E–1G). However, it was surprising that TBF pretreatment significantly alleviated the phenotype described above (Figure 1G, 1H). Figure 1M shows the statistical analysis of the results of the H&E staining of the mammary gland (Figure 1M, *p* < 0.01).

By analyzing the results of Masson staining, we found that compared with the NT group and the TBF group, there were more woven collagen fibers in the HFD group, as indicated by the arrow in the blue area (Figure 1I–1K). This result implies that a HFD induced fibrosis in the mammary gland. However, it was surprising that TBF pretreatment significantly alleviated the phenotype described above (Figure 1K, 1L). The TBF group alone did not show damage to the mammary tissue morphology, which indicated that the concentration of TBF selected was safe in this experiment. The key time nodes and related information of this experimental cycle were displayed in the form of a time axis (Figure 1N).

### The effect of TBF on inflammatory levels induced by HFD during pregnancy and lactation

**Figure 2:**
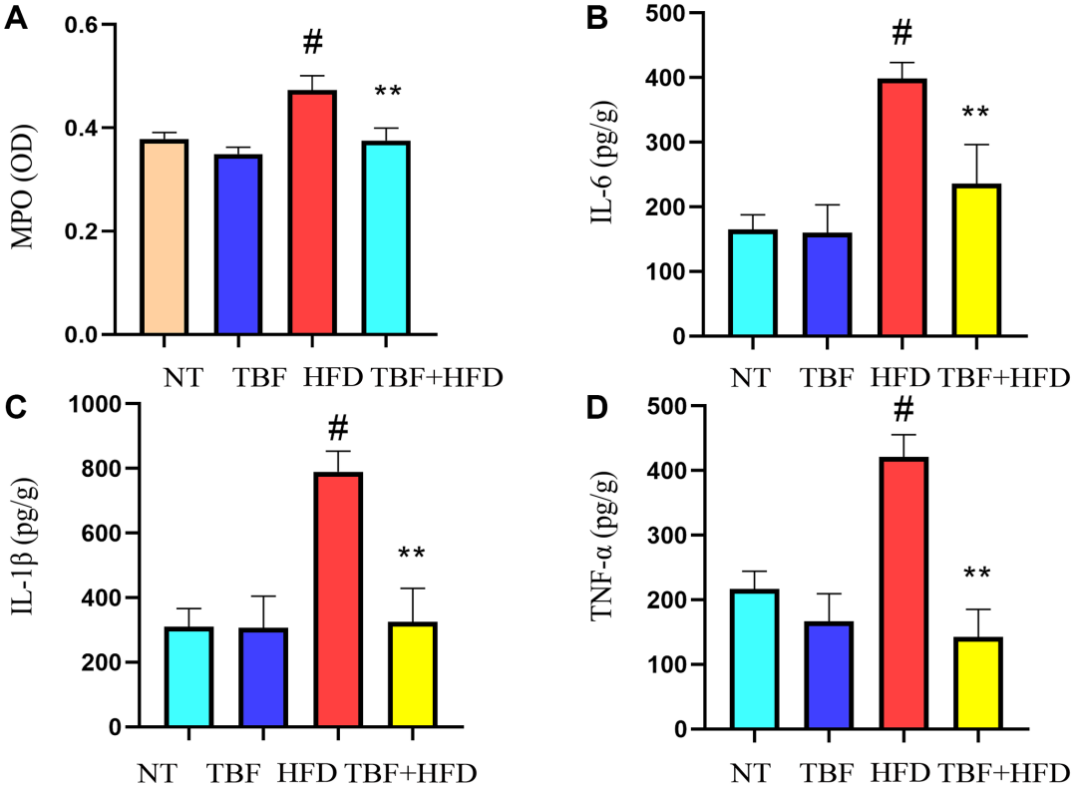
**The effect of TBF on inflammatory levels induced by HFD during pregnancy and lactation.** 1 g/L TBF was dissolved in 0.1% carboxymethylcellulose sodium solution and was ingested by mice by drinking water. Drinking water administration began on the first day of mating and stopped one week after delivery, and then mammary tissue was collected. After grinding and centrifugation, the supernatant of mice mammary tissue was obtained, and then the levels of MPO and pro-inflammatory cytokines in mammary tissue were determined. (**A**) Changes in MPO levels in mammary tissue; (**B**) Changes in IL-6 levels in mammary tissue; (**C**) Changes in IL-1β levels in mammary tissue; (**D**) Changes in the level of TNF-α in mammary tissue; The data error was based on SEM. Three independent repeated experiments were performed; ^#^*p* < 0.01 vs. NT group; ^∗∗^*p <* 0.01 vs. HFD group.

To explore the influence of a HFD on the inflammatory microenvironment of the mammary gland during pregnancy and lactation and to determine whether TBF intake through drinking water has an inhibitory effect on inflammation of the mammary gland, we assessed changes in mammary tissue inflammation-related indicators. Our experimental results showed that myeloperoxidase (MPO) level in the HFD group was significantly increased compared with that in the NT group and the TBF group (Figure 2A, *p* < 0.01) and that the secretion of inflammatory cytokines (IL-6, IL-1β, TNF-α) was also significantly higher (Figure 2B–2D, *p* < 0.01). However, it was interesting that drinking water containing TBF significantly alleviated the phenotype described above (Figure 2A–2D, *p* < 0.01). These results indicated that TBF alleviates the tendency toward HFD-induced fibrosis of mammary tissue by reducing the levels of inflammatory mediators in mammary tissue.

### Effect of TBF on AKT/NF-κB signaling induced by HFD during pregnancy and lactation

**Figure 3:**
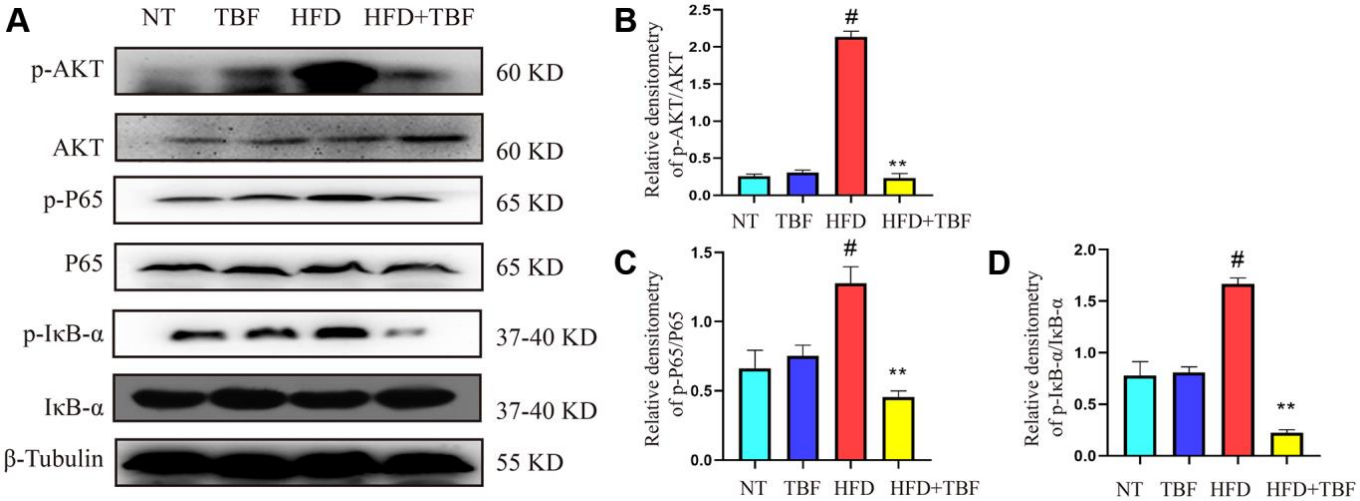
**The effect of TBF on AKT/NF-κB signal induced by HFD during pregnancy and lactation.** 1 g/L TBF was dissolved in 0.1% carboxymethylcellulose sodium solution and was ingested by mice by drinking water. Drinking water administration began on the first day of mating and stopped one week after delivery, and then mammary tissue was collected. (**A**) Protein bands of p-AKT, p-P65, p-IκB, AKT, P65, IκB, β-Tublin; (**B**) Analysis of relative density value of p-AKT protein; (**C**) Analysis of relative density value of p-P65 protein; (**D**) Analysis of the relative density value of p-IκB protein. The data error was based on SEM, three independent repeated experiments were performed; ^#^*p* < 0.01 vs. NT group; ^∗∗^*p <* 0.01 vs. HFD group.

To explore the potential mechanism by which TBF alleviates the damage to mammary tissue caused by HFD during pregnancy and lactation, we examined the changes in AKT/NF-κB in mammary tissue from the perspective of inflammation. Our results showed that the phosphorylation level of AKT protein in the HFD group was significantly higher than that in the NT group (Figure 3A, 3B, *p* < 0.01). In addition, the downstream signal of AKT, NF-κB, was also significantly activated and specifically expressed as the P65 and IκB proteins (Figure 3A, 3C, 3D, *p* < 0.01). However, it was interesting that drinking water containing TBF significantly inhibited the activation of the AKT/NF-κB signaling pathway (Figure 2A–2D, *p* < 0.01). This result indicated that TBF inhibits the level of inflammation in mammary tissue by inhibiting the activation of the AKT/NF-κB signaling pathway.

### Molecular dynamics simulation of rutin and quercetin and AKT protein

**Figure 4:**
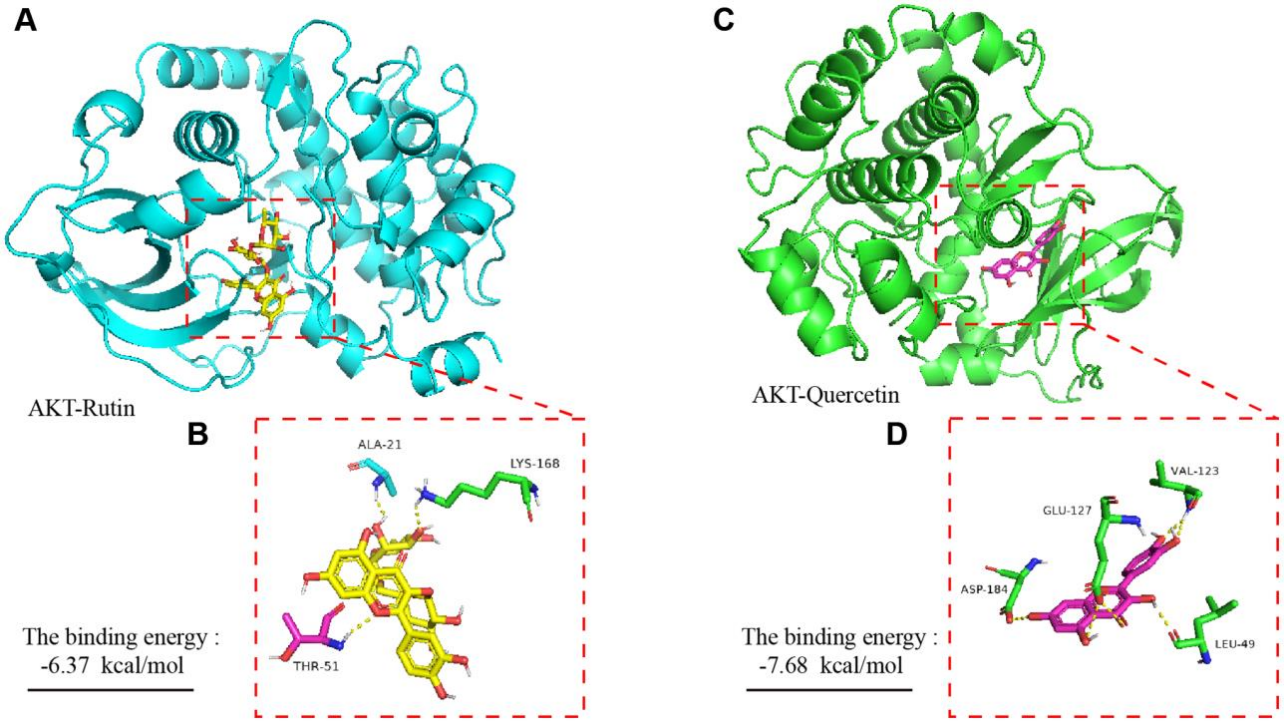
**Molecular dynamics simulation of TBF and AKT protein.** (**A**) The spatial binding mode of Rutin and AKT protein. (**B**) Amino acid residues where Rutin and AKT protein have hydrogen bonds, ALA-21, LYS-168, THR-51. (**C**) The spatial binding mode of Quercetin and AKT protein. (**D**) Quercetin and AKT protein have hydrogen bonding amino acid residues, VAL123, GLU-127, ASP-184, LEU-49. The 3D structure of the AKT protein was obtained from the PDB database with the number 3ow3. TBF contains 36.3% rutin and 58.2% quercetin. The 3D structure of rutin and quercetin came from PubChem, and then stored in PyMOL as pdbqt mode. AutoDock 4 was used to simulate the molecular docking of AKT with rutin and quercetin. The simulation was obtained by the software AutoDock 4, and the molecular docking pattern is obtained by the software PyMOL. The number of molecular docking simulations was performed 2.5 × 10^6^.

To explore the potential interaction sites of rutin and quercetin with the AKT protein, we used bioinformatics technology (AutoDock 4) to simulate the molecular docking between the small molecules and the protein. Our experimental results showed that rutin formed hydrogen bonds with the amino acid residues THR-51, ALA-21 and LYS-168 of the AKT protein (Figure 4A, 4B), and the sum energy between them was −6.37 kal/mol, which is statistically significant when the sum energy is less than 0. Similarly, quercetin showed hydrogen bonding with the amino acid residues ASP-184, GLU-127, VAL-123, and LEU-49 of AKT (Figure 4C, 4D). In summary, the sum energy was −7.68 kal/mol, and the bond energy value was relatively large, which was statistically significant.

### The effect of TBF on the blood-milk barrier induced by HFD during pregnancy and lactation and its underlying mechanism

In order to further explore the potential mechanism of TBF alleviating the tendency of mammary fibrosis, we tested the effects of TBF and HFD on the blood-milk barrier and the expression of related tight junction proteins from the perspective of the blood-milk barrier.

**Figure 5:**
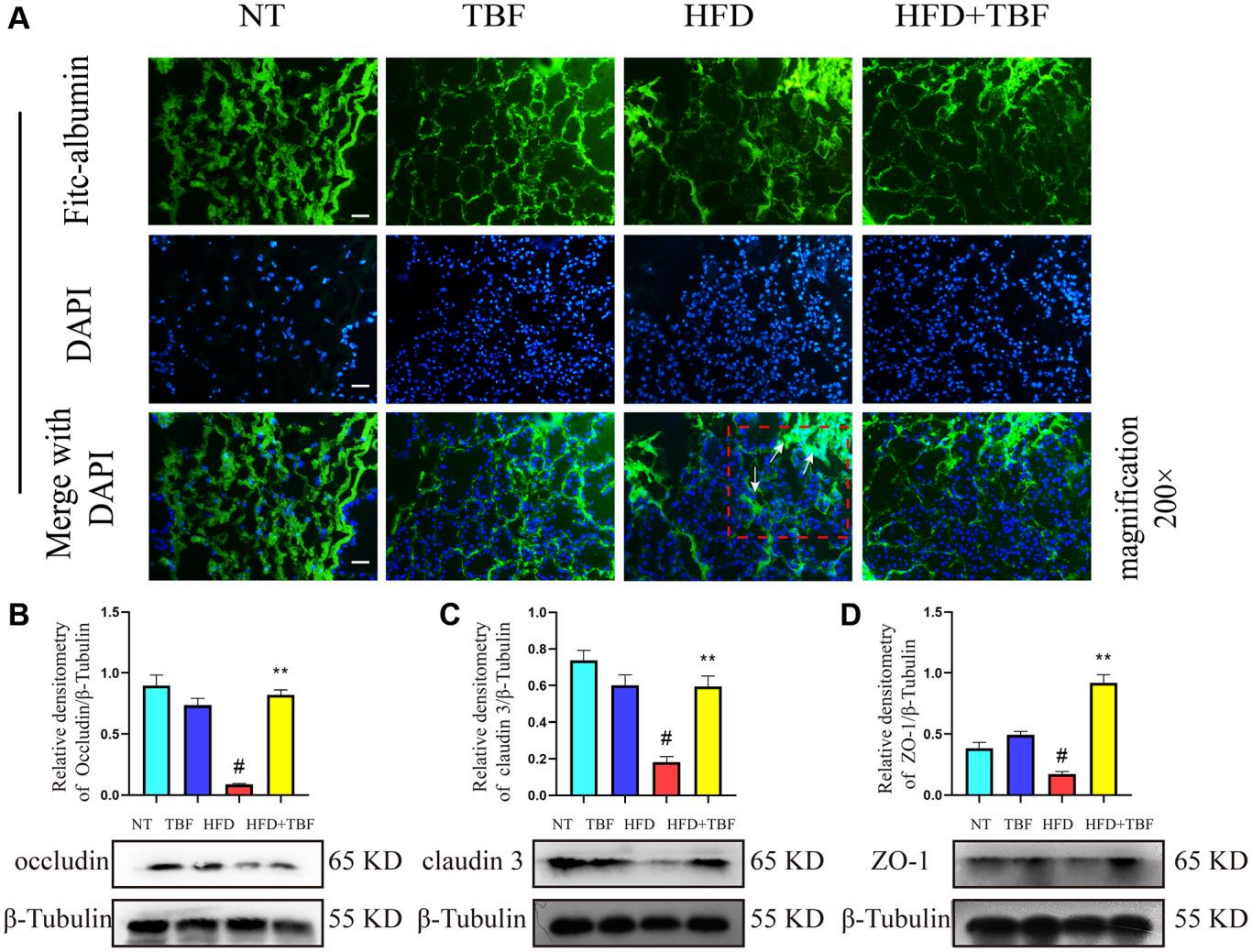
**The effect of TBF on the blood-milk barrier induced by HFD during pregnancy and lactation.** Fresh mice mammary tissue was immersed in 2 mg/mL FITC for 15 min, then the mammary tissue block was placed in liquid nitrogen, and finally the tissue block was frozen sectioned by a cryostat. After DAPI staining, the sections were sealed with anti-fluorescence quenching reagent, finally obtain the experimental results. FITC green fluorescent protein shuttle freely between the fresh mammary acinar interstitiumed and leaked into the damaged mammary acinar. In addition, FITC will show large green fluorescence in the mammary tissue with acinar atrophy matrix thickening. The lesion was shown by the arrow in the figure, and the scale bar is 100 μm. (**A**) Flow trajectory of FITC solution in mammary gland; (**B**) Occludin protein band and relative density analysis results; (**C**) Analysis results of the band and relative density value of Claudin-3 protein; (**D**) ZO-1 protein band and relative density analysis results; β-Tublin was used as a reference for protein control. The data error was based on SEM, three independent repeated experiments were performed; ^#^*p* < 0.01 vs. NT group; ^∗∗^*p* < 0.01 vs. HFD group.

Our results showed that the integrity of the mammary barrier of the HFD group was damaged compared with that of the NT group, which was manifested by the leakage of FITC solution flowing in the interstitial space into the acinus. In addition, the thickening of the stroma between the alveoli showed large clusters of green FITC fluorescence, and the arrow indicates the specific lesion (Figure 5A). However, it was gratifying that the phenotype mentioned above were significantly alleviated in the mice after drinking water containing TBF during pregnancy and lactation and that the integrity of the blood-milk barrier was significantly repaired (Figure 5A).

In addition, our research results also showed that the expression of the tight junction proteins occludin, claudin-3, and ZO-1 in the HFD group was significantly lower than that in the NT group. These results suggested that a HFD during pregnancy and lactation destroyed the mammary glands. The weakened tight junction structure between epithelial cells led to the destruction of the blood-milk barrier, leaving the mammary gland in a state of being stimulated by external factors for a long time. However, it was gratifying that after administration of drinking water containing TBF during pregnancy and lactation, the effects described above were significantly alleviated and the tight junction structure was repaired (Figure 5B–5D, *p* < 0.01).

### The effect of TBF on fibrosis induced by HFD during pregnancy and lactation

The above results suggest that TBF may inhibit the process of HFD-induced mammary fibrosis by inhibiting the mammary gland inflammatory microenvironment and protecting the integrity of the blood-mammary barrier. To further verify whether a HFD can cause mammary fibrosis and whether TBF alleviates HFD-induced mammary fibrosis, fibrosis markers were assessed.

**Figure 6:**
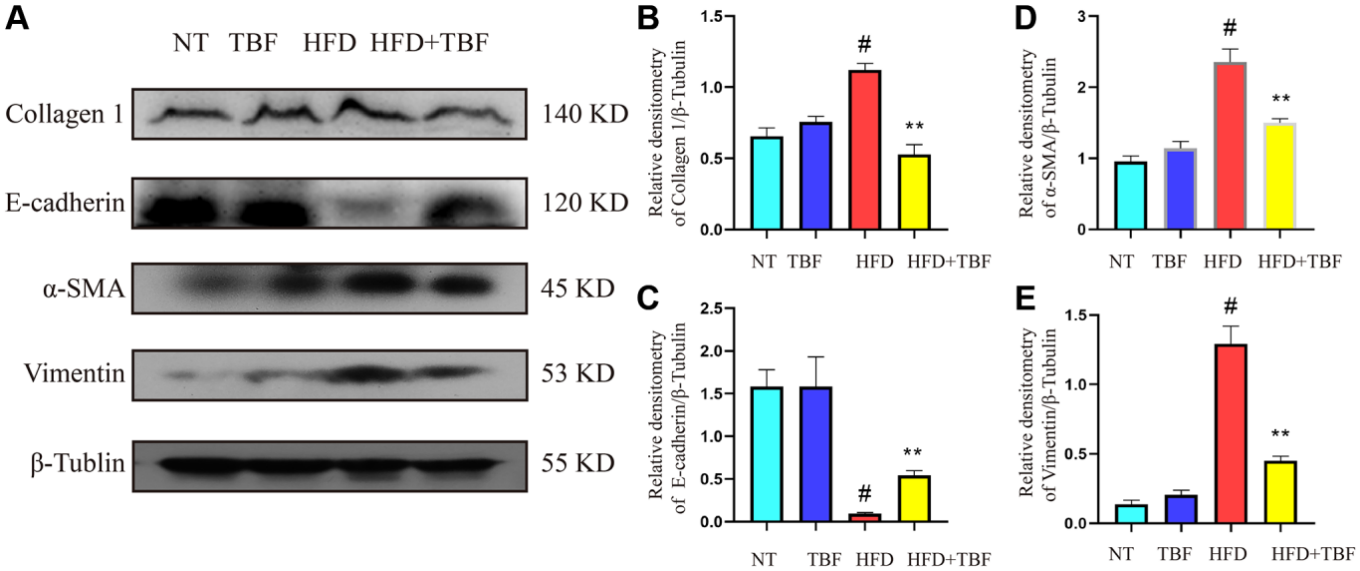
**The effect of TBF on fibrosis induced by HFD during pregnancy and lactation.** 1 g/L TBF was dissolved in 0.1% carboxymethylcellulose sodium solution and was ingested by mice by drinking water. Drinking water administration began on the first day of mating and stopped one week after delivery, and then mammary tissue was collected. (**A**) Collagen 1, E-cadherin, α-SMA, Vimentin, β-Tublin protein bands; (**B**) Analysis of the relative density value of Collagen 1 protein; (**C**) Analysis of the relative density value of E-cadherin protein; (**D**) Analysis of relative density value of α-SMA protein; (**E**) Analysis of the relative density value of vimentin protein; β-Tubulin was used as a reference for protein control. The data error was based on SEM, three independent repeated experiments were performed; ^#^*p* < 0.01 vs. NT group; ^∗∗^*p* < 0.01 vs. HFD group.

Our results showed that the expression of E-cadherin in the HFD group was significantly lower than that in the NT group (Figure 6A, 6C, *p* < 0.01), suggesting that the polarity and integrity of mammary epithelial cells were damaged; the expression of collagen 1 increased significantly (Figure 6A, 6B, *p* < 0.01), suggesting that the extracellular matrix (ECM) in the mammary tissue of the HFD group was produced in large quantities, which provided a growth environment for mammary tissue fibrosis. In addition, the expression of vimentin and α-SMA in the HFD group was significantly increased (Figure 6A, 6D, 6E, *p* < 0.01), suggesting that fibroblasts in mammary tissue were activated and could secrete ECM in large quantities. These results confirmed that a HFD during pregnancy and lactation led to a more serious tendency toward fibrosis in mammary tissue. However, it was gratifying that drinking TBF in advance during pregnancy and lactation significantly inhibited the expression of vimentin, α-SMA, and collagen 1, promoted the expression of E-cadherin (Figure 6A, 6E, *p* < 0.01), and relieved the occurrence of fibrosis in mammary tissue.

### The effect of TBF on TGF-β/Smad signaling induced by HFD during pregnancy and lactation

The above results suggest that on the one hand, HFD-induced inflammation and destruction of the blood-milk barrier can lead to the occurrence of breast fibrosis; on the other hand, HFD can also directly induce the occurrence of breast fibrosis. TBF can act on these signals at the same time to play a protective role. To further verify the potential mechanism by which TBF alleviates HFD-induced mammary fibrosis, TGF-β/Smad signaling was assessed.

**Figure 7:**
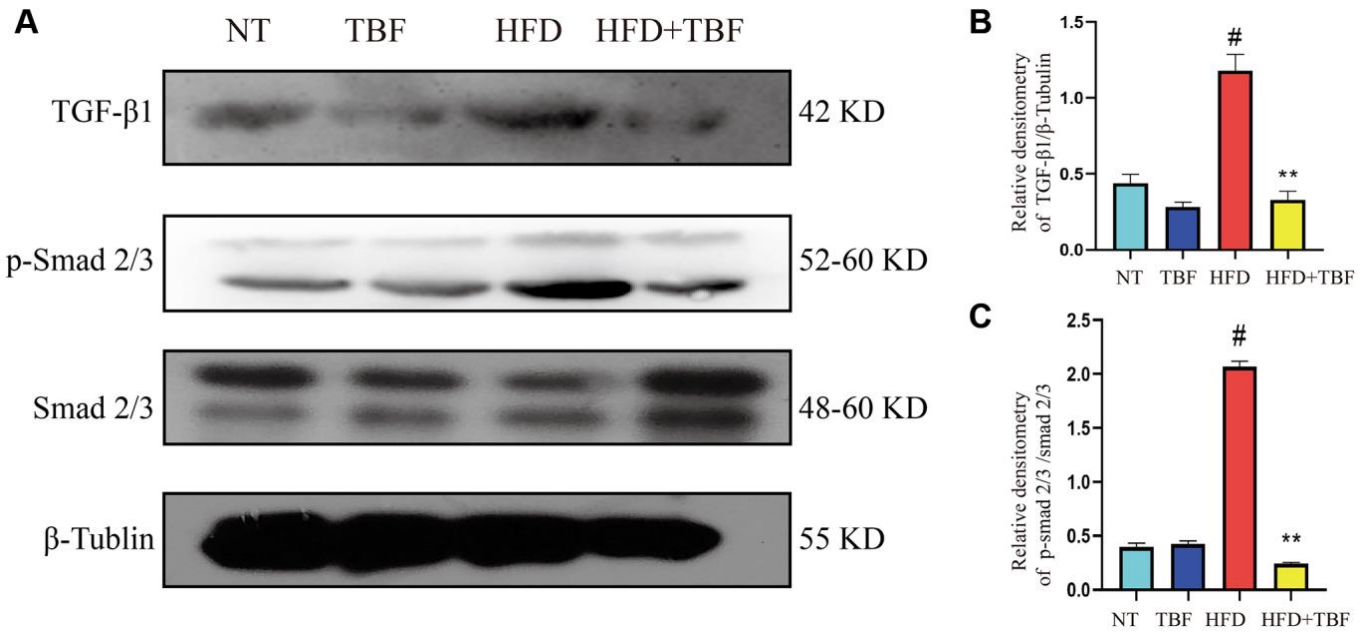
**The effect of TBF on TGF-β/Smad signal induced by HFD during pregnancy and lactation.** 1 g/L TBF was dissolved in 0.1% carboxymethylcellulose sodium solution and was ingested by mice by drinking water. Drinking water administration began on the first day of mating and stopped one week after delivery, and then mammary tissue was collected. (**A**) Protein bands of TGF-β1, p-Smad2/3, Smad2/3, β-Tubulin; (**B**) Analysis of the relative density value of TGF-β1 protein; (**C**) Analysis of relative density value of p-Smad2/3 protein; β-Tublin was used as a reference of TBF-β1, Smad2/3 was used as a reference of p-Smad2/3. The data error was based on SEM, three independent repeated experiments were performed; ^#^*p* < 0.01 vs. NT group; ^∗∗^*p* < 0.01 vs. HFD group.

Our results showed that the expression level of TGF-β1 in the HFD group was significantly higher than that in the NT group (Figure 7A, 7B, *p* < 0.01), suggesting that there is a large amount of TGF-β1 in the mammary gland that stimulates the normal mammary tissue to transition to fibrosis. In addition, the phosphorylation level of Smad 2/3 was significantly increased (Figure 7A, 7C, *p* < 0.01), suggesting that a large amount of Smad 3 and its complexes translocate into the nucleus to promote the expression of a large number of genes associated with fibrosis, which maintain and promote the transformation process of fibrosis. However, it was gratifying that drinking TBF in advance during pregnancy and lactation significantly inhibited the activation of the TGF-β/Smad signaling pathway (Figure 7A–7C, *p* < 0.01) and alleviated mammary fibrosis.

### Molecular dynamics simulation of rutin and quercetin and Smad 3 MH2 domains of TBF group

**Figure 8:**
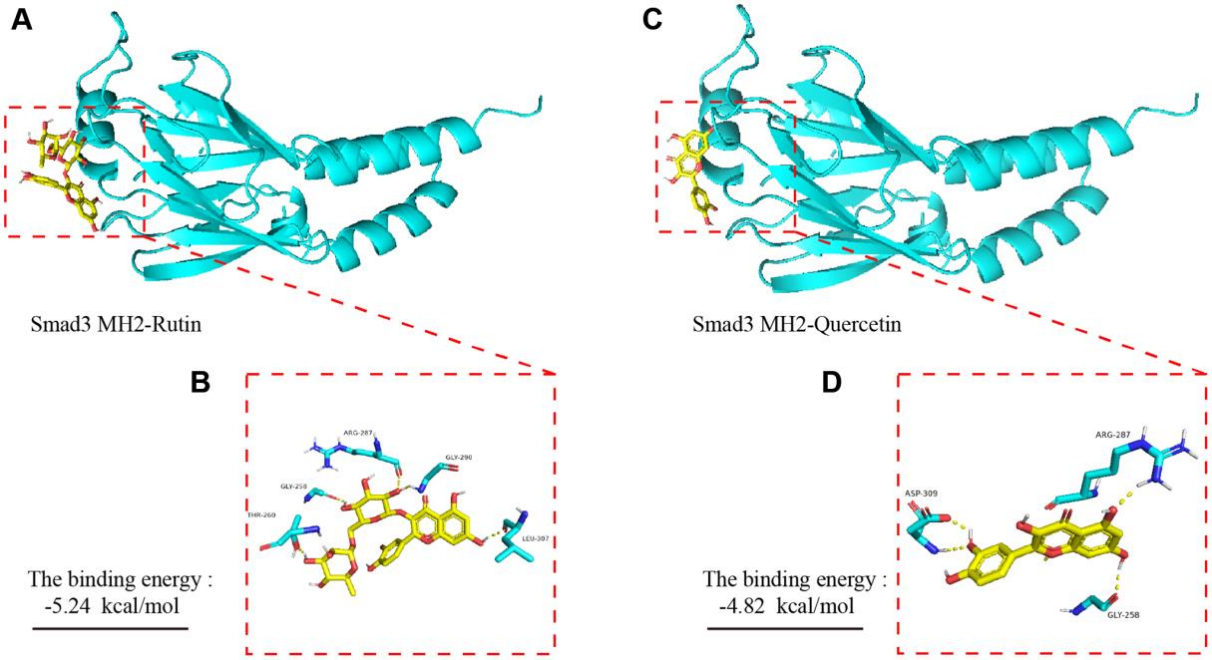
**Molecular dynamics simulation of TBF and Smad 3 MH2 domain.** (**A**) The spatial binding mode of Rutin and Smad3 MH2 domain protein. (**B**) Rutin and MH2 domain protein have hydrogen bonding amino acid residues, ALA-21, LYS-168, THR-51. (**C**) The spatial binding mode of Quercetin and MH2 domain protein. (**D**) Quercetin and MH2 domain protein have hydrogen bonding amino acid residues, VAL123, GLU-127, ASP-184, LEU-49. The 3D structure of the Smad 3 MH2 domain is obtained from the PDB database, and the number is 1MJS. TBF contains 53.6% rutin and 37.2% quercetin. The 3D structure of rutin and quercetin came from PubChem, and then stored in PyMOL as pdbqt mode. AutoDock 4 was used to simulate the molecular docking of AKT with rutin and quercetin. The simulation is obtained by the software AutoDock 4, and the molecular docking pattern is obtained by the software PyMOL. The number of calculations for molecular docking simulation is executed 2.5 × 10^6^.

To explore the potential interaction sites of rutin and quercetin (the main component of rutin) with the MH2 domain of Smad 3, we applied bioinformatics technology (AutoDock 4) to simulate the molecular docking of the small molecules with the proteins. Our experimental results showed that there was hydrogen bonding between rutin and the amino acid residues ARG-287, GLY-290, GLY-258, THR-260 and LEU-307 of the MH2 domain of Smad 3 (Figure 8A, 8B). The total binding energy was −5.24 kal/mol, and the sum energy was statistically significant when it was less than 0. Similarly, the amino acid residues of quercetin and ARG-287, ASP-309, and GLY-258 of AKT showed hydrogen bonding (Figure 8C, 8D). The sum energy was −4.82 kal/mol, which was statistically significant.

## DISCUSSION

Mammary disease is a disease with a serious pathological process and complicated influencing factors [22, 23]. Finding ways in which to prevent mammary disease and protect mammary health is a problem to be solved. Our previous research results indicated that a HFD during pregnancy shapes the mammary gland microenvironment and destroys the blood-milk barrier [1, 23]. It is thought-provoking to consider whether HFD during pregnancy and lactation will cause irreversible damage to the mammary glands and whether eating certain natural products can alleviate irreversible damage to the mammary gland. The experimental results showed that TBF significantly alleviated the tendency toward fibrosis in mammary tissue caused by HFD during pregnancy and lactation, and the underlying mechanism was explored in-depth.

Inflammatory mediators are the main components of the inflammatory microenvironment [24], and long-term stimulation of the inflammatory microenvironment is the main stimulating factor that induces tissue fibrosis, tissue sclerosis, and tissue cancer [25]. Long-term infiltration of inflammatory mediators in mammary tissue increases cell sensitivity, reduces cell autoimmunity, and reduces the ability to regulate the homeostatic balance of mammary tissue [26, 27]. On the other hand, inflammatory mediators can stimulate the closure of the mammary duct, resulting in milk deposition in the mammary gland, further aggravating the adverse factors of stimulating mammary tissue [28]. Our research results showed that TBF significantly alleviated the increase in IL-6, IL-1β, TNF-α, and MPO levels in mammary tissue and effectively controlled the formation of the inflammatory microenvironment. This is consistent with the current research idea of protecting mammary health by controlling the level of mammary inflammatory mediators [29, 30].

AKT is an important pro-inflammatory protein kinase. Phosphorylation of AKT transmits inflammatory signals downstream, thereby promoting the formation of an inflammatory microenvironment and exacerbating the long-term stimulation of inflammation in mammary tissue [31]. Therefore, AKT is often used as a potential target for the treatment of inflammatory diseases. For example, studies have shown that inhibition of AKT phosphorylation can effectively alleviate the mast cell-mediated inflammatory response [32]. NF-κB is a classic and very important inflammatory signaling pathway located downstream of AKT and is composed of the IκB and P65 subunits [33]. After NF-κB is phosphorylated by AKT, P65 translocates into the nucleus to promote the release of inflammatory cytokines. Studies have shown that inhibiting the phosphorylation of NF-κB can relieve mastitis [29, 34]. Our results showed that TBF significantly inhibits the activation of NF-κB in mammary tissue induced by HFD.

The blood-milk barrier is an important biological barrier of the mammary gland, and the integrity of the blood-milk barrier is an important guarantee for its normal physiological functions [35, 36]. Studies have shown that the blood-milk barrier is destroyed during mastitis, which increases the risk of mammary gland exposure, reduces the ability of the mammary gland to resist infection by pathogenic microorganisms and forces the mammary gland to be in the stimulation state of an inflammatory environment for a long time [35, 37, 38]. In addition, studies have shown that the destruction of the lung barrier is closely related to idiopathic pulmonary fibrosis [7]. Studies have shown that the destruction of tight junction structures leads to the loss of cell polarity and promotes epithelial mesenchymal transition. However, epithelial mesenchymal transition is an important pathological process that promotes fibrosis [9]. Therefore, repairing the tight junction structure and maintaining the normal physiological function of the barrier may be a potential therapeutic approach for fibrotic diseases. Our previous study showed that a HFD during pregnancy can destroy the blood-milk barrier [1]. However, whether a HFD during pregnancy and lactation induces fibrosis of the mammary gland by destroying the blood-milk barrier has not yet been reported. Our research results showed that TBF significantly alleviated the inhibition of the expression of the tight junction proteins occludin, claudin-3, and ZO-1 and the destruction of the blood-milk barrier by HFD feeding and helped prevent HFD-induced fibrosis of the mammary gland.

The large accumulation of ECM is the main feature of tissue fibrosis [39]. Studies have shown that inhibiting the secretion of collagen 1 alleviates liver fibrosis [40]. While the ECM is mainly secreted by activated fibroblasts, vimentin and α-SMA are the main signs of fibroblast activation [41, 42]. Therefore, inhibiting the levels of vimentin and α-SMA is a potential treatment for alleviating fibrosis [41, 42]. E-cadherin is the basis for maintaining epithelial cells to ensure the normal structure and physiological functions of epithelial cells [43]. Decreased expression of E-cadherin promotes the migration ability of fibroblasts and aggravates the process of fibrosis [43]. Therefore, promoting the expression of E-cadherin is an effective means to alleviate mammary fibrosis. Our results showed that TBF significantly inhibited the expression of collagen 1, vimentin and α-SMA while promoting the expression of E-cadherin.

TGF-β/Smad is a classical signaling pathway in fibrosis [44]. Studies have shown that the expression level of TGF-β1 in fibrotic tissues is significantly higher than that in normal tissues [44]. Smad 3 is regulated by TGF-β1, an important transcription factor that promotes fibrosis [40, 44]. TGF-β1 promotes the phosphorylation of Smad 3 to form a Smad complex, which translocates into the nucleus to activate fibroblasts, promotes the production of ECM, destroys cell polarity, and aggravates the process of fibrosis [41, 44]. Studies have shown that inhibiting the activation of TGF-β/Smad is an important target for inhibiting the occurrence of fibrosis [40, 41]. Our research results indicated that TBF significantly inhibits the tendency of a HFD to induce mammary fibrosis during pregnancy and lactation.

## CONCLUSIONS

**Figure 9:**
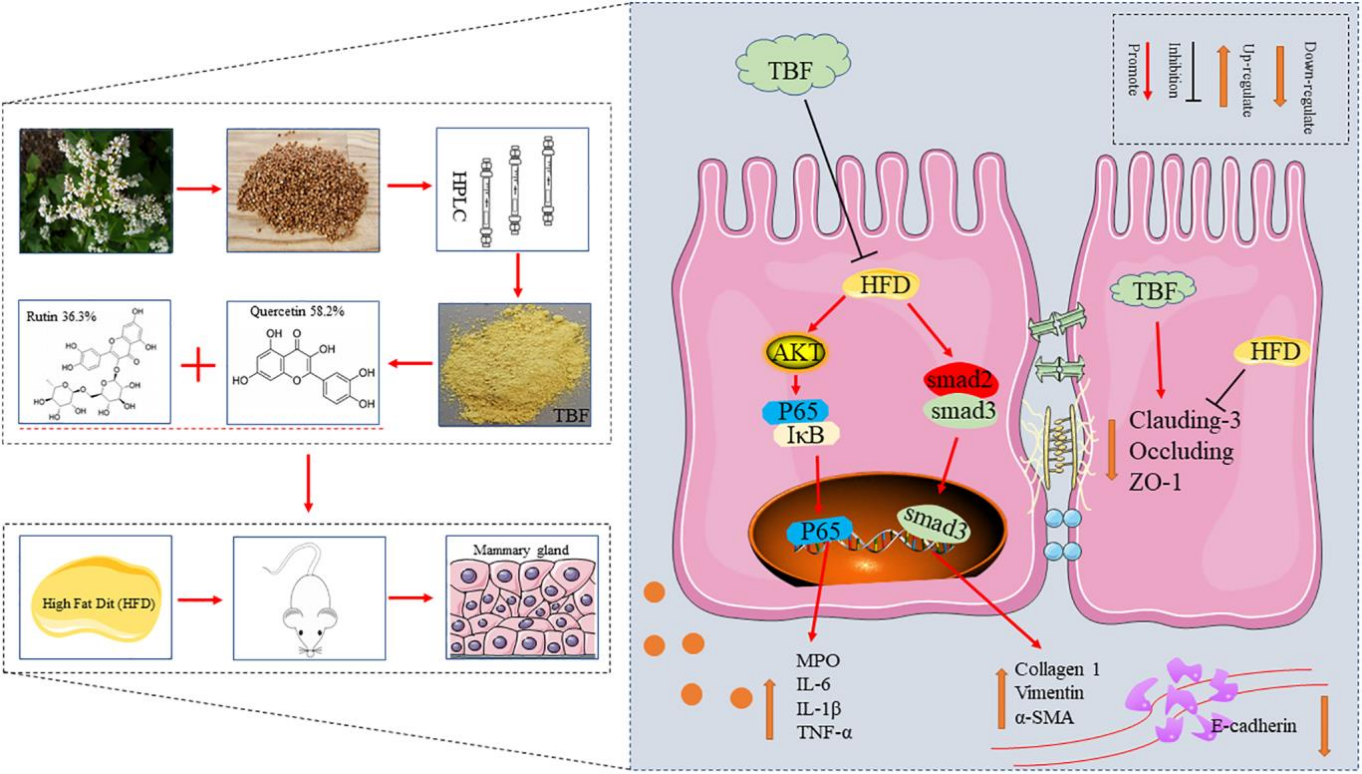
**The effect of TBF on alleviating mammary fibrosis tendency and its underlying mechanism induced by HFD during pregnancy and lactation.** HFD during pregnancy and lactation caused inflammation of mammary tissue, destroyed the blood-milk barrier, and induced fibrotic lesions tendency in mammary. TBF significantly alleviates the inflammatory response caused by HFD, repairs the blood-milk barrier, and relieves the tendency of mammary tissue fibrosis. Potential protective mechanism of TBF: TBF inhibited the activation of the AKT/NF-KB signaling pathway, effectively inhibiting the inflammatory response to the induction of fibrosis; TBF promoted the expression of tight junction proteins claudin-3, occluding, and ZO-1, and repairs blood-milk barrier to reduce the risk of mammary tissue being exposed; TBF inhibited the activation of TGF-β/Smad signal and inhibits the occurrence of fibrosis tendency.

This study demonstrated that HFD in pregnancy and lactation induced a tendency for mammary fibrosis and that the intake of drinking water containing TBF played a significant role in alleviating mammary fibrosis by inhibiting the activation of AKT/NF-κB signaling pathway, controlling the long-term induction of inflammatory microenvironment, repairing the blood-milk barrier and reducing the stimulation of external factors. These results indicate that inhibiting the activation of the TGF-β/Smad signaling pathway and preventing the transformation of fibrosis are potential protective mechanisms of TBF (Figure 9).
